# Hypertensive disorders of pregnancy & vascular dysfunction

**DOI:** 10.3389/fcvm.2024.1411424

**Published:** 2024-05-30

**Authors:** Anna Palatnik, Jacquelyn Kulinski

**Affiliations:** ^1^ Division of Maternal Fetal Medicine, Medical College of Wisconsin, Milwaukee, WI, United States; ^2^ Division of Cardiovascular Medicine, Medical College of Wisconsin, Milwaukee, WI, United States

**Keywords:** preeclampsia, vascular dysfunction, flow-mediated dilation, biomarkers, cardiovascular risk

## Abstract

Hypertensive disorders of pregnancy (HDP) are a leading cause of maternal and fetal morbidity and mortality. One of the more severe HDP diagnoses is preeclampsia, which is recognized as a sex-specific cardiovascular risk enhancer with long-term implications for women's health, increasing lifetime risk of ischemic heart disease, stroke, and heart failure. Though the mechanisms accounting for the increased risk of cardiovascular disease following HDP are not yet well understood, vascular dysfunction has been implicated. In this perspective piece, we summarize the existing evidence for vascular dysfunction in HDP with a focus on non-invasive assessments, highlight advances in the field, and suggest future directions for improving risk stratification of women with HDP.

## Introduction

Hypertensive disorders of pregnancy (HDP) are a heterogenous group of diagnoses which include chronic hypertension, gestational hypertension, and preeclampsia with and without severe features (1). HDP have become more common over the past 3 decades, affecting approximately 15% of women during their reproductive years and are a leading cause of maternal and fetal morbidity and mortality (2–5). Delivery hospitalization data for 2017–2019 analyzed from the National Inpatient Sample, a nationally representative sample of all U.S. hospital discharges, showed that among maternal deaths during the index (delivery) hospitalization, 31.6% had any HDP (6).

One of the more severe HDP diagnoses is preeclampsia. Preeclampsia is a multisystem progressive disorder characterized by the new onset of hypertension and proteinuria or the new onset of hypertension plus significant end-organ dysfunction with or without proteinuria, presenting after 20 weeks of gestation or postpartum (1). Preeclampsia is a sex-specific cardiovascular risk enhancer with long-term implications for women's health, with an associated increase in lifetime risk of ischemic heart disease, stroke, and heart failure, often early in onset (7). Data from a national registry in Denmark linked preeclampsia to a 4-fold increased risk of heart attack and 3-fold increased risk of stroke within the first decade after delivery compared to women without a history of preeclampsia (8). Cardiovascular risk further increases with severity of preeclampsia symptoms, preeclampsia onset at earlier gestational age, and with increasing number of pregnancies complicated by preeclampsia (9). However, other than screening for and managing traditional cardiovascular disease (CVD) risk factors, little guidance exists on how to appropriately risk-stratify and treat women with a prior history of preeclampsia.

The mechanisms that account for increased risk of CVD following HDP are not yet well understood but could be related to vascular dysfunction (10). Endothelial dysfunction is thought to have a critical role in preeclampsia and may represent a mechanistic link to future CVD. Vessels obtained from the soft tissue of women with preeclampsia show compromised endothelium-dependent dilation while maintaining endothelium-independent dilation (11). Furthermore, vascular dysfunction persists for years after a hypertensive pregnancy with studies showing lower flow-mediated dilation and higher arterial stiffness in those with HDP compared to controls (12, 13). In this perspective piece, we will summarize the existing evidence for vascular dysfunction in HDP with a focus on non-invasive assessments, highlight advances in the field, and suggest future research directions for improving risk stratification of women with HDP.

## Brachial artery flow-mediated dilation (BA-FMD)

Flow-mediated dilation of the brachial artery (BA-FMD) has become the most widely used technique to measure endothelial function, using the forearm circulation as a surrogate for coronary arteries (14). It is a non-invasive measurement of conduit vessel vascular endothelial function, measured as the change in brachial artery diameter after a hyperemic flow stimulus. The technique measures the ability of the arteries to respond with endothelial nitric oxide (*N*O) release during reactive hyperemia. BA-FMD is a strong predictor of future cardiovascular health and has been shown to be abnormal in HDP. BA-FMD is a well-established method of evaluating future cardiovascular disease risk in research settings though not in the clinical setting. BA-FMD predicts cardiovascular events in healthy populations and in patients with established CVD whereby a 1% increase in BA-FMD indicates a significant 8%–13% lower risk of cardiovascular events (15, 16).

We now have longitudinal data on maternal vascular endothelial function from early pregnancy to delivery and postpartum. A meta-analysis including 37 studies examined BA-FMD before, during, or after preeclampsia (13). When compared with women who did not have preeclampsia, women who had preeclampsia had lower FMD before the development of preeclampsia, around 20–29 weeks gestation, with overall standardized mean difference in FMD of −0.84 (−1.19, −0.50); at the time of preeclampsia [−1.41 (−2.00, −0.83)], and for up to three years postpartum [−0.90 (−1.26, −0.54)] (13). Similar results were observed even after exclusion of women with chronic hypertension and/or smokers. A history of preeclampsia did not have a significant effect on FMD when assessed approximately 10 years postpartum, although this analysis should be interpreted with caution, as it was limited to only four cross-sectional studies with moderate heterogeneity (13). These results support the concept that vascular dysfunction precedes the onset of preeclampsia and may contribute to its pathophysiology. Whether persistent vascular dysfunction is related to risk factors pre-dating pregnancy or is a direct result of lasting damage to the heart and vasculature remains unclear. Perhaps persistent vascular dysfunction could identify those women who might be more suitable for more intensive or newer therapeutic approaches to mitigate future CV risk.

## Peripheral arterial tonometry (PAT)

The different physiological roles of conduit and resistance arteries should be considered. Whereas reduced NO release to stimuli underlies endothelial dysfunction in the conduit arteries (BA-FMD), NO in the microcirculation may primarily modulate tissue metabolism (17). Digital peripheral arterial tonometry (PAT) is a non-invasive method to assess microvascular endothelial function, using arterial pulsatile volume changes at the fingertip, in response to a hyperemic flow stimulus, commonly expressed as the reactive hyperemia index (RHI). It reflects changes in flow, as well as in digital microvessel dilatation and is only partly dependent on NO (18). Vascular dysfunction is defined as an RHI ≤ 1.67. PAT response has been associated with the presence of obstructive and nonobstructive coronary artery disease (19, 20) and correlates with coronary microvascular function (21). Interestingly, BA-FMD may be particularly sensitive to impairment by traditional risk factors (e.g., age, hypertension), whereas the PAT reactive hyperemia index (RHI) of the microvasculature may be more sensitive to metabolic risk factors, such as body mass index and diabetes mellitus (17). Furthermore, PAT associates only modestly with BA-FMD, thus likely measuring different aspects of vascular biology. As such, it is suggested that both BA-FMD and PAT should both be evaluated whenever possible.

Fewer investigators have used PAT as an assessment of endothelial dysfunction in studies of preeclampsia. One such study enrolled 180 women with at least two risk factors for preeclampsia at gestational weeks 16 and 28, of which 24 women developed preeclampsia or pregnancy-induced hypertension. There was no difference in RHI between cases and controls at either week 16 or 28 or at 6–9 months postnatally (22). These investigators questioned the reliability of PAT measurements later in pregnancy, when women are more vasodilated. In a separate study by Orabona, et al., RHI was examined between 6 months and 4 years after delivery in women without previous preeclampsia (*n* = 30) or with early-onset (*n* = 30) or late-onset (*n* = 30) preeclampsia. RHI was only impaired in those women with the early-onset preeclampsia [37% of women with abnormal RHI ≤ 1.67 (mean RHI 1.70 ± 0.42)]. RHI was within normal range in late-onset preeclampsia though was significantly lower compared to controls (mean RHI 2.51 ± 0.49 v. 2.89 ± 0.35, *p* < 0.05) (23). All included women were free of traditional CV risk factors and drugs at the time of exam. Additionally, all women with any preeclampsia exhibited increased arterial stiffness (see the next section). Given the paucity of studies evaluating PAT in women with HDP, we do not currently recommend routine use of PAT for identification of these high-risk women during pregnancy.

## Arterial stiffness and augmentation

Studies demonstrated that arterial stiffness and augmentation are significantly higher in HDP compared to normotensive pregnancy (12, 24, 25). Pulse wave velocity (PWV), a measure of arterial stiffness, is calculated as the distance traveled by the pulse wave divided by the time taken to travel the distance. PWV can be measured in any arterial segment between two pulse-wave palpable regions, such as between the carotid and femoral arteries. Increases in the propagation speed of the pulse indicate increases in arterial stiffness. A meta-analysis of 17 longitudinal studies including 15,877 subjects demonstrated that an increase of carotid-femoral PWV by 1 m/s corresponds to an adjusted risk increase of 14% in total vascular events after mean follow-up of 7.7 years (26).

Similarly, arterial pulse waveforms in peripheral arteries, such as the radial artery, can be measured non-invasively by applanation tonometry to generate an augmentation index (AIx). Radial AIx has been reported to show a close correlation with aortic AIx (*r *= 0.81–0.96), suggesting a similar physiological significance between aortic and radial AIx (27). Since this parameter is influenced by heart rate, it is often standardized (ex. per 75 bpm shown as AIx75). A growing body of evidence has indicated that arterial stiffness is more closely or independently correlated with future cardiovascular events than is brachial blood pressure (28, 29). Vascular compliance is known to improve during normal healthy pregnancies. Augmentation index declines during pregnancy, reaches its nadir in mid-pregnancy and then rises towards term (27). In the previously mentioned study by Orabona, et al., peripheral AIx75 was increased in both early-onset and late-onset preeclampsia, though more so in the former, compared to controls (17 ± 9% v. 6 ± 13% v. −2 ± 6%; intergroup ANOVA < 0.001) (23), suggesting reduced arterial compliance. A consistent finding of 14 studies of women with HDP was an increase in cfPWV and AIx prior to disease onset, during and up to 2–3 years postpartum (30). This supports the concept that arterial stiffness precedes the development of preeclampsia. Therefore, these measurements may enable earlier identification of high-risk HDP populations that would benefit from earlier CVD risk factor management.

## Carotid intima-media thickness (CIMT)

Ultrasound measurement of the combined thickness of the intima and media layers of the carotid artery (CIMT) in the neck has been used as a tool to detect early stages of atherosclerosis prior to a clinical cardiovascular event. Several large, research-based cohort studies have clearly indicated a relationship between CIMT and CVD events (31–33). CIMT values over 0.9 mm (European Society of Cardiology) (34) or over the 75th percentile (American Society of Echocardiography) (32) are considered abnormal.

A 2017 systematic review and meta-analysis by Milic, et al., included 14 studies in women with preeclampsia (35). Seven studies were conducted during pregnancy complicated by preeclampsia and 10 studies were carried out up to 10 years postpartum (3 studies included measurements both during and after the pregnancy) (35). Women with preeclampsia had significantly higher CIMT than did those who did not have preeclampsia, both at time of diagnosis [standardized mean difference (SMD), 1.10 (95% CI, 0.73–1.48; *p* < 0.001)] and in the first decade postpartum [SMD, 0.58 (95% CI, 0.36–0.79); *p* < 0.001]. The effect remained significant in a sensitivity analysis that excluded women with chronic hypertension at the time of their pregnancies. There were not enough studies to determine whether women who develop preeclampsia have higher CIMT values prior to preeclampsia diagnosis.

More recently (2020), in a study of 220 pregnant women with CIMT measured every 3 months during pregnancy, CIMT values were significantly higher in patients who developed preeclampsia (36). Using a cut-off value of 0.51 mm, CIMT had a specificity of 77.9% and sensitivity of 81% in the diagnosis of preeclampsia. With CIMT ≥0.6 mm, the probability of a patient developing preeclampsia was 44.4%; with CIMT >0.42 mm, the probability was only 4.2% (36). Therefore, CIMT could potentially be useful in the identification of high-risk women during pregnancy.

Current guidelines do not support routine measurement of CIMT in CVD risk assessment for the general population (37). This recommendation is based on evidence provided by Den Ruijter et al, that the addition of CIMT measurements to the Framingham Risk Score was associated with a small and clinically non-significant improvement in 10-year prediction of the first atherosclerotic CVD event (38). Additional rationale for the recommendation included concerns about measurement quality in addition to different consensuses for measurement of CIMT. However, as the interest in risk prediction is currently shifting from a 10-year risk to lifetime risk, the added value of a CIMT measurement using a horizon of 20–30 years may warrant additional exploration (38), especially in highly selected and younger patient sub-groups, such as women with HDP.

## Uterine artery flow

The placenta is thought to have a significant role in the pathophysiology of preeclampsia. Spiral arteries are the maternal uterine arteries leading to the placenta. The acute atherosis that characterizes the spiral arteries during preeclampsia is similar to the early stages of atherosclerosis (39). The term “acute” refers to the fact that these lesions develop over a relatively short time period (during the pregnancy) and may also disappear rapidly after delivery (40). This acute atherosis is characterized by subendothelial lipid-filled foam cells, vascular (fibrinoid) necrosis, and perivascular lymphocyte infiltration (41). The inadequate maternal uterine spiral artery remodeling is thought to cause a dysfunctional uteroplacental circulation with oxidative stress and augmented generation of factors released into the maternal circulation, leading to an excessive maternal inflammatory response and endothelial dysfunction (42). The decreased uterine blood flow leads to placental ischemia. Uterine artery doppler ultrasound in the first trimester appears to be a highly specific test for the prediction of early preeclampsia with moderate sensitivity. A large meta-analysis studied the detection rate of abnormal uterine artery pulsatility index in the first trimester of low-risk women, showing a specificity of 92.1% and sensitivity of 47.8% for a false positive rate of 8% (43). In a study of sixty-two high-risk patients followed throughout gestation at a large, academic medical center, all underwent Doppler velocimetry of the uterine arteries. Ten of these pregnancies were complicated by early-onset preeclampsia, and these patients had a significantly higher pulsatility index of the uterine arteries between 16- and 19-weeks' gestation (prior to the diagnosis of preeclampsia), compared with the normotensive group (44). Because there is limited evidence that an accurate prediction of early-onset preeclampsia can be followed by interventions that improve maternal or fetal outcomes, the American College of Obstetricians recommends that use of uterine artery Doppler studies remain investigational at this time (as of 2020) (1).

## Serum biomarkers in preeclampsia

Two placenta-derived angiogenic biomarkers, soluble fms-like tyrosine kinase 1 (sFlt-1) and placental growth factor (PIGF) have proved useful as diagnostic and prognostic tests for preeclampsia. sFlt-1 is secreted from placental trophoblast cells into maternal circulation. Circulating sFlt-1 adheres to the receptor-binding domains of vascular endothelial growth factor (VEGF) and PIGF (a VEGF homolog), preventing their interaction with endothelial cells, inducing endothelial dysfunction (45). In addition to endothelial dysfunction, high levels of sFlt-1 result in vasoconstriction and immune dysregulation, negatively impacting multiple maternal organ systems and the fetus (46). sFlt-1 mRNA is increased (and PIGF proportionately decreased) in placentas of individuals with preeclampsia, and serum sFlt-1 levels are almost five times higher in severe preeclampsia compared with normotensive pregnancies (47). This discovery was followed by a number of studies using the sFlt-1/PIGF ratio to diagnose preeclampsia, and then also to predict preeclampsia and adverse outcomes. In INSPIRE (Interventional Study Evaluating the Short-term Prediction of Preeclampsia/Eclampsia), a randomized trial of 370 women presenting with suspected preeclampsia, use of the test improved hospitalization to 100% of patients who developed preeclampsia within 7 days (compared to only 83% of those without the result revealed) (48). Cost savings were observed in all studies across multiple countries evaluating the economic impacts of implementing the sFlt-1/PIGF ratio test and were attributed to improved diagnostic accuracy and a reduction in unnecessary hospitalization (49). In May 2023, the U.S. Food and Drug Administration approved a biomarker screening test (sFlt-1/PIGF) at 24–34 weeks of gestation, shown to have a 94% sensitivity and 75% specificity, to identify patients at high risk of severe preeclampsia.

Levels of sFlt-1 rapidly decrease post-partum, confirming that it is almost entirely derived from the placenta. Unfortunately, measurement of sFlt-1, PGIF or their ratio measured during HDP was not found to be predictive of hypertension 1 year postpartum (50).

## Discussion

Pregnancy complicated by preeclampsia is associated with systemic vascular dysfunction. A systematic review and meta-analysis pooling results from 72 studies in 8,702 women, demonstrated vascular dysfunction in women after HDP compared with women with prior normal pregnancy when measured by carotid-femoral pulse wave velocity [0.64 m/s (0.17–1.11)], carotid intima–media thickness [0.025 mm (0.004–0.045)], and augmentation index [5.48% (1.58–9.37)], as well as mean levels of soluble fms-like tyrosine kinase [6.12 pg/ml (1.91–10.33)] (12). Between group differences were more pronounced when assessments were performed in younger women (<40 years) or closer to the index pregnancy for almost all modalities. Pooled analyses were not conducted for PAT due to fewer than 3 available studies. The totality of evidence supports some persistent vascular dysfunction after HDP. With a modest mean difference of 12%, sFlt-1 was the only biomarker consistently higher in women with prior preeclampsia relative to women with recent normotensive pregnancy (12).

Vascular imaging with BA-FMD appears to be a useful tool to identify women with vascular dysfunction both early in pregnancy and in the postpartum period after HDP. In contrast, sFlt-1 can identify patients at high risk of severe preeclampsia during pregnancy but when measured after HDP, is not as sensitive in identifying underlying endothelial damage. Calculated 10-year CV risk assessments in these women, such as with the pooled cohort equation from the American Heart Association, are often falsely low due to young age and lack of integration of HDP history. Similarly, waiting for hard CV outcomes in pragmatic clinical trials of HDP would yield low event rates. Though traditionally used in the context of research, clinical use of vascular imaging modalities, such as BA-FMD, in the postpartum period might help further define an at-risk group to be targeted for more aggressive risk factor modification (12). Whether more aggressive blood pressure control early postpartum in HDP ameliorates vascular dysfunction, and thus CVD risk, is a provocative question that needs to be tested and could have important implications for the future cardiovascular care of these women.

## Data Availability

The original contributions presented in the study are included in the article/Supplementary Material, further inquiries can be directed to the corresponding author.
